# Impact of neoadjuvant therapy on cancer stem cell gene expression and miRNA profiles in breast cancer patients: Implications for therapy resistance and survival, a cross-sectional observational study

**DOI:** 10.1097/MD.0000000000043077

**Published:** 2025-06-27

**Authors:** Tuba Dilay Kökenek Ünal, Müzeyyen Burcu Kaplan Yilmaz, Olçay Kandemir, Samet Yora, Serpil Taheri

**Affiliations:** a Betul Ziya Eren Genome and Stem Cell Center, Erciyes University, Kayseri, Turkey; b Department of Pathology, Faculty of Medicine, Ankara Yildirim Beyazit University, Ankara, Turkey; c Department of Pathology, Abdurrahman Yurtaslan Onkoloji Research and Training Hospital, Ankara, Turkey; d Department of Pathology, Gülhane Faculty of Medicine, University of Health Sciences, Ankara, Turkey; e Department of Molecular Biology and Genetics, Faculty of Medicine, Erciyes University, Kayseri, Turkey.

**Keywords:** breast cancer stem cells, *CD24*, *CD44*, miR399-3p, miR590-3p, miR599

## Abstract

Breast cancer is one of the most common cancers in women worldwide. Several therapy modalities have been created recently; however, resistance to therapy is a major issue. Cancer stem cell functions and regulations are important in tumor progression, invasion, metastasis, and therapy resistance. The expression levels of cancer stem cell genes *CD44*, *CD24*, and related miRNAs miR590-3p, miR599, and miR399-3p were aimed to be investigated before and after neoadjuvant therapy in breast cancer patients in this cross-sectional observational study. This study included 80 samples from 40 female patients. The expression of *CD44* and *CD24* genes and miR590-3p, miR599, and miR399-3p was analyzed by qPCR in pre- and posttreatment biopsies from breast carcinoma patients. Correlations between expression levels and other pathologic parameters, including molecular subtypes, grade, stage, metastasis, recurrence, pathologic response to therapy, and disease-free and overall survival, were investigated. *CD44* and *CD24* mRNA expression levels decreased significantly after treatment. However, miR590-3p expression increased after treatment. Patients with complete pathologic responses had upregulated *CD24* and downregulated miR590-3p and miR399-3p levels in initial biopsies. Univariate analysis showed that increased expression levels of miR590-3p, miR599, and miR399-3p were significantly associated with shorter disease-free survival. A better understanding of the role of cancer stem cells in cancer can result in more promising results and patient-tailored therapy options. This study highlights the significant value of cancer stem cells and related miRNAs in response to therapy and recurrence.

## 1. Introduction

Breast cancer is the most common cancer among women worldwide with a slow rise in its incidence.^[1]^ Its clinical management differs from other cancers due to its molecular heterogeneity and the relative predictability of treatment response. Several clinicopathological parameters assessed at initial diagnosis provide valuable prognostic information. However, further research is needed to identify more comprehensive biomarkers for accurate risk stratification and outcome prediction. Breast cancer is classified into distinct biological subtypes, underscoring the importance of personalized therapeutic approaches. Neoadjuvant therapy is increasingly utilized in select patient cohorts, demonstrating significant clinical benefits. Investigating the precise mechanisms underlying its favorable impact on tumor biology and treatment outcomes remains a critical research priority.^[2]^

Various therapy modalities have been developed for breast carcinoma in recent years, but chemoresistance is an important problem in achieving successful treatment. Current therapies have been targeting the bulk of the tumor, and the possible potential of breast cancer stem cells (CSC) has been ignored.^[3]^ CSCs are a small subset of cancer cells with significant roles in tumor initiation and development, metastasis, therapy resistance, and recurrence.^[4]^ Recent anticancer therapies reduce tumor cells, but CSCs survive and proliferate because of an enhanced de novo or acquired resistance to these medications. This renders the clinical treatment useless, promotes tumor recurrence, and raises the death rate.^[5]^

The expression of stemness biomarkers is also an important biomarker predicting prognosis and survival in cancers, including breast carcinoma.^[6]^ Stemness biomarkers refer to a set of molecular markers that are functionally associated with the biological properties of CSCs. These include self-renewal capacity, differentiation potential, tumor initiation, and therapy resistance. Operationally, stemness biomarkers for breast cancer were identified through prior experimental evidence such as *ALDH1*, *CD44*, *CD24*, and were evaluated in patient samples to correlate with clinical outcomes or treatment responses.^[7]^ We previously studied *ALDH1A1* and *CD133* expressions in breast carcinoma and found that *CD133* is a promising marker for predicting treatment response.^[8]^

miRNAs have emerged as critical molecules because of their prognostic or therapeutic importance. In recent years, a burgeoning body of research has delved into applying compounds that mimic or inhibit miRNAs in cancer therapy.^[9]^ The ongoing research on miRNAs as a novel therapeutic target highlights their increasing importance in cancer treatment. This study investigates the molecular expression of selected CSC markers (*CD44*, *CD24*, miR590-3p, miR599, and miR339-3p) in breast carcinoma patients before and after neoadjuvant chemotherapy (NAC). It was hypothesized that the response to therapy would be reflected in the expressions of CSC markers. The potential prognostic and predictive value of genes and miRNAs in breast cancer patients was aimed to be explored.

## 2. Materials and methods

### 2.1. Case selection

The study included 80 samples of 40 female patients with invasive breast carcinoma. The biopsy samples and surgical materials after NAC were selected from the archive of the Pathology Department of Dr Abdurrahman Yurtaslan Oncology Research and Training Hospital (Ethics committee approval ID 2019-08/354, 07.08.2019 by the hospital’s ethics committee). The study included female patients aged 20 to 80 with invasive breast carcinoma, provided their first diagnostic biopsy samples were available and they were undergoing NAC. Exclusion criteria included male patients, female patients with other subtypes of breast cancer, patients with synchronous or metachronous cancer and patients which were undergoing therapy for another cancer.

After reevaluation of all slides, suitable paraffin blocks were selected for molecular investigation.

Response to the therapy was evaluated, and the tumor regression score was calculated according to online Residual Cancer Burden Calculator (https://www3.mdanderson.org/app/medcalc/index.cfm?pagename=jsconvert3). Patients having a pathologic complete response (pCR) were regarded as responsive to the therapy, and patients having a near complete response, partial response, and poor or no response were regarded as nonresponders.

Based on the immunohistochemical expression of estrogen (ER), progesterone (PR), and human epidermal growth factor (HER2), patients were categorized into clinical/molecular subtypes as ER+/Luminal B (ER+), HER2+/HER2-enriched (HER2+), and triple negative/basal-like (TN).^[10]^ The other parameters such as tumor size, nuclear grade, Ki67 index, lymph node (LN) metastasis, distant metastasis, response to treatment, stage, recurrence, and disease-free and overall survival were documented.

### 2.2. RNA isolation, cDNA synthesis, and qPCR analysis

RNA isolation was performed from the paraffin blocks using the RNeasy FFPE Kit for RNA Extraction (Qiagen, Germany). cDNA synthesis was performed from total RNA using the RT2 First Strand Kit (Qiagen, Germany) for mRNA expression and miRCURY LNA RT Kit for miRNA expression. The mRNA expressions of *CD44* (Entrez Gene ID: 960, QT00073549, QuantiTect Primer Assays, Qiagen, Germany) and *CD24* (Entrez Gene ID: 100133941, QT00216811, QuantiTect Primer Assays, Qiagen, Germany) genes were evaluated by quantitative PCR and FastStart™ Universal SYBR® Green Master (Roche, Nederland) on Rotor-Gene Q (Qiagen, Germany). The Actin beta gene (Entrez Gene ID: 60, QT00095431, QuantiTect Primer Assays, Qiagen, Germany) for mRNA was used as a control. Secondly, miRNA expression levels were determined using specially designed primers for miR590-3p (MIMAT ID: MIMAT0004801, Sequence UAAUUUUAUGUAUAAGCUAGU, YP00205448, Qiagen, Germany), miR599 (MIMAT ID: MIMAT0003267, Sequence GUUGUGUCAGUUUAUCAAAC, YP00205908, Qiagen, Germany), miR339-3p (MIMAT ID: MIMAT0004801, Sequence UCCCUGUCCUCCAGGAGCUCACG, YP00206007, Qiagen, Germany), and miRCURY LNA SYBR Green PCR Kits. U6 SnRNA (YP02119464, Qiagen, Germany) was used as a control. The concentration and purity of RNA were assessed using a NanoDrop 2000 (Thermo Fisher Scientific, MA). Only RNA samples exhibiting A260/A280 and A260/230 ratio of 1.8 to 2.0 and a concentration above 10 ng/μL were included for further analysis. The cycling conditions were set as follows: an initial denaturation at 95°C for 2 minutes, followed by 40 cycles of denaturation at 95°C for 15 seconds and annealing/extension at 56°C for 1 minute. A melting curve program was subsequently performed from 60°C to 95°C. The qPCR was repeated twice, and Ct values were used in a delta-delta-Ct (2-∆∆Ct) analysis.

### 2.3. Statistical analysis

Statistical analysis was performed by SPSS 22.0 for Windows (SPSS, Inc.; Chicago). Descriptive statistics were defined as number (n), percentage (%), mean ± standard deviation, and median. The normal distribution of data is evaluated using histogram, q–q plot, and Shapiro–Wilk test. Pearson chi-square and Fisher’ s exact test were used to compare categorical parameters. Willcoxon rank test was used for comparison of dependent groups and the Mann–Whitney *U* test or Kruskal–Wallis test was used for comparison of independent groups. The *P*-values from the Kruskal–Wallis test were adjusted using the Bonferroni correction test. Chi-square was used for categorical parameters. The Spearman correlation test was used to evaluate the relationship of variables. *P* < .05 was considered statistically significant.

## 3. Results

The study included a total of 40 female patients with a range of 28 to 74 (mean 51) years old. The ER + group included 20 patients, and HER2 + and TN groups contained 10 patients each. There were 10 patients with ER+/PR+/HER2 + immune profile and 10 patients with ER+/PR+/HER2‐ immune profile in ER + group. Twenty-three patients (57.5%) responded well to the therapy, but 17 patients (42.5%) showed poor or no response.

The follow-up period was 5 years. Recurrence was observed in 6 patients (4 in TN, 1 in ER+, and 1 in HER2+), and 3 patients (all in the TN group) died in this period. The 3 patients with recurrent disease had a pCR and the death was observed in 1 patient with pCR. Disease-free survival (DFS) was 7 to 89 months (mean 50 months). Overall survival (OS) was 9 to 89 months (mean 54 months). All descriptive data was summarized in Table 1.

**Table 1:** Patient characteristics.

		Total
Age		
	Under 45	15 (37%)
	Over 45	25 (63%)
Tumor size		
	<2 cm	6 (15%)
	2–5 cm	22 (55%)
	>5 cm	12 (30%)
Localization of tumor		
	Right	20 (60%)
	Left	16 (40%)
Hormone status		
	Positive	20 (50%)
	Negative	20 (50%)
cerbB2 status		
	Positive	20 (50%)
	Negative	20 (50%)
Lymph node metastasis		
	Present	31 (77%)
	Absent	9 (23%)
Distant metastasis		
	Present	9 (23%)
	Absent	31 (77%)
Stage		
	Stage I	1 (3%)
	Stage II	13 (32%)
	Stage III	20 (50%)
	Stage IV	6 (15%)
Residual cancer burden		
	Grade 0	21 (53%)
	Grade 1	2 (5%)
	Grade 2	11 (27%)
	Grade 3	6 (15%)
Recurrence		
	Present	6 (15%)
	Absent	34 (85%)
Survival		
	Alive	37 (92%)
	Deceased	3 (8%)

We found no statistically significant differences among clinical/molecular subtypes in initial biopsies in response to therapy.

Tumor size ranges between 13 and 100 mm (median 38.90). We found that the larger tumor size is significantly related to the probability of recurrence (*P* = .017). The levels of *CD44* and *CD24* were correlated (*P* = .01), and miR590-3p, miR599, and miR399-3p were correlated with each other (*P* = .001). The obtained data do not indicate a significant relationship between miRNAs and genes.

### 3.1. The expression of *CD44* is significantly decreased after neoadjuvant chemotherapy

The expression of the *CD44* gene decreased significantly after therapy (*P* = .001) (Table 2). The decrease in *CD44* expression level by therapy was significant in ER + and TN groups (*P* = .0001 and *P* = .017, respectively) (Table 3). Moreover, the cerbB2-negative tumors had significantly decreased levels of *CD44* after treatment (*P* = .001). There was a significant positive correlation between cerbB2 and *CD44* gene expressions (*P* = .008). However, the *CD44* levels did not differ between hormone-positive and negative tumors.

**Table 2:** The pretreatment and posttreatment levels of *CD44*, *CD24*, miR590-3p, miR599, and miR399-3p in breast cancer patients.

	Pretreatment levels median (IQR)	Posttreatment levels median (IQR)	*P*
*CD44*	0.91335 (0.04922–5.28302)	0.00880 (0.00225–0.24605)	**.001**
*CD24*	0.90970 (0.055975–19.75180)	0.09465 (0.01602–0.50940)	**.001**
miR590-3p	0.45885 (0.19922–1.18005)	1.73270 (0.15970–6.76862)	**.042**
miR599	0.44465 (0.07822–6.25930)	5.32785 (0.24255–19.3466)	.265
miR399-3p	0.67385 (0.17270–3.60825)	0.64245 (0.17525–5.86552)	.554

Statistically significant *P* values are represented in bold.

**Table 3:** The pretreatment and posttreatment levels of *CD44*, *CD24*, miR590-3p, miR599, and miR399-3p in breast cancer subtypes.

Total	ER+		HER2+		TN	
	Pretreatment median (IQR)	Posttreatment median (IQR)	Pretreatment median (IQR)	Posttreatment median (IQR)	Pretreatment median (IQR)	Posttreatment median (IQR)
*CD44*	0.91950 (0.04922–8.00625)	0.004950 (0.00152–0.20665)	0.44925 (0.14592–1.42330)	0.15445 (0.02152–9.6990)	1.33275 (0.03335–75.71175)	0.00440 (0.00150–0.045300)
	**.0001**		.721		**.017**	
*CD24*	0.90970 (0.07142–4.15887)	0.47150 (0.00945–0.15080)	15.17175 (0.00672–103.46285)	10.46735 (0.43747–116.11697)	0.34140 (0.04480–43.55717)	0.09225 (0.04047–0.24130)
	**.002**		.721		**.047**	
miR590-3p	0.68210 (0.31010–1.20660)	2.71510 (0.86510–17.04170)	0.21070 (0.12275–7.11260)	0.31490 (0.00905–8.14630)	0.29655 (0.20462–1.91695)	1.00255 (0.00250–4.38010)
	**.017**		.678		1.00	
miR599	0.37140 (0.07822–16.85380)	5.3777 (0.23535–19.3665)	0.90920 (0.09097–6.25930)	3.83480 (0.20847–182.40807)	0.34435 (0.06642–31.32437)	4.63900 (1.42727–11.9798)
	.681		.285		.575	
miR399-3p	1.12815 (0.11190–5.76890)	0.78040 (0.02742–12.94682)	0.67385 (0.18920–9.100425)	0.91730 (0.018250–10.95602)	0.55100 (0.46532–2.23847)	0.01790 (0.00532–2.82207)
	.601		.959		.646	

Statistically significant *P* values are represented in bold.

The *CD44* levels in patients with distant metastasis were significantly higher than in nonmetastatic cases at initial biopsies (*P* = .033). There was an upregulation of the *CD44* in patients with LN metastasis and high-stage tumors in initial biopsies compared to the patients without LN involvement and low stage tumors, but the differences were insignificant. On the contrary, the pretreatment levels of *CD44* in recurrent cases were significantly lower than in nonrecurrent cases (*P* = .033). *CD44* levels in initial biopsies didn’t differ significantly among molecular subtypes.

The expression levels of *CD44* in patients with pCR significantly differed between pre- and posttreatment biopsies (*P* = .02) despite no difference in nonresponders (Table 4). The patients with pCR showed a higher level of expression of *CD44* in pretreatment biopsies than those without pCR, but it was not significant. When we consider clinical subtypes, we observed a significant decrease in *CD44* levels in patients with pCR in ER + group after therapy (*P* = .008) (Table 5). In HER2 + and TN groups, the difference in *CD44* expression was not significant (Tables 6 and 7).

**Table 4:** The alteration of gene and miRNA levels in patients with pathologic complete response and with no response by neoadjuvant chemotherapy.

Total	Response to therapy	Pretreatment levels median (IQR)	*P* ^1^	Posttreatment levels median (IQR)	*P* ^2^
*CD44*	pCR	1.17610 (0.11515–8.27905)	.350	0.00900 (0.26695–38.01905)	**.002**
	No pCR	0.21260 (0.32300–1.71000)		0.00860 (0.00150–1.22010)	.117
*CD24*	pCR	2.17050 (0.26695–38.01905)	**.044**	0.05320 (0.14950–0.37715)	**.001**
	No pCR	0.07480 (0.00860–2.58110)		0.19260 (0.03660–1.80130)	.445
miR590-3p	pCR	0.26255 (0.09275–0.83732)	**.014**	1.95530 (0.00792–7.60560)	**.011**
	No pCR	0.810050 (0.448525–4.50202)		1.31065 (0.22330–6.36182)	.638
miR599	pCR	0.18170 (0.06585–2.41970)	.060	5.27800 (0.08170–113.59280)	.073
	No pCR	5.1777 (0.09920–16.85380)		5.37770 (0.41640–9.36320)	.687
miR399-3p	pCR	0.51190 (0.11530–1.83420)	**.027**	0.37220 (0.1765–7.93215)	.339
	No pCR	1.50940 (0.27430–17.43600)		0.69690 (0.01420–6.18600)	.059

Statistically significant *P* values are represented in bold.

pCR = pathologic complete response, *P*^1^ = *P* values indicate the difference in pretreatment level of parameters between patients with pathologic complete response and nonresponders, *P*^2^ = *P* values indicate the difference between pre- and posttreatment levels of parameters in patients with pathologic complete response and nonresponders.

**Table 5:** The pre- and posttreatment expression levels of *CD44*, *CD24*, miR590-3p, miR599, and miR399-3p by pathologic response in ER + subtype of breast carcinoma.

ER+	Response to therapy	Pretreatment levels median (IQR)	*P* ^1^	Posttreatment levels median (IQR)	*P* ^2^
*CD44*	pCR	0.99580 (0.17780–5.86305)	.732	0.00560 (0.00260–0.30245)	**.008**
	No pCR	0.61010 (0.00490–563.33850)		0.00430 (0.00130–0.05350)	.013
*CD24*	pCR	1.45300 (0.37200–5.07070)	.138	0.01550 (0.00650–0.04870)	**.008**
	No pCR	0.07480 (0.01860–2.58110)		0.10220 (0.00910–0.29480)	.248
miR590-3p	pCR	0.35000 (0.08060–1.12840)	.105	3.09985 (0.62975–14.60640)	**.017**
	No pCR	0.69160 (0.58160–2.01110)		2.39160 (1.06510–37.21980)	.237
miR599	pCR	0.12910 (0.04450–0.36920)	**.017**	5.37770 (0.05275–319.84615)	.110
	No pCR	5.3777 (0.29060–48.73880)		5.37770 (0.25260–9.36320)	.286
miR399-3p	pCR	0.15060 (0.34100–2.28500)	.063	0.86390 (0.03395–24.59360)	.441
	No pCR	1.50940 (0.17360–17.43600)		0.69690 (0.00910–6.25060)	.131

Statistically significant *P* values are represented in bold.

pCR = pathologic complete response, *P*^1^ = *P* values indicate the difference in pretreatment level of parameters between patients with pathologic complete response and nonresponders, *P*^2^ = *P* values indicate the difference between pre- and posttreatment levels of parameters in patients with pathologic complete response and nonresponders.

**Table 6:** The pre- and posttreatment expression levels of *CD44*, *CD24*, miR590-3p, miR599, and miR399-3p by pathologic response in HER2+ subtype of breast carcinoma.

HER2+	Response to therapy	Pretreatment levels median (IQR)	*P* ^1^	Posttreatment levels median (IQR)	*P* ^2^
*CD44*	pCR	1.30680 (0.49220–1438367.491)	.117	0.03600 (0.01735–4.41395)	.500
	No pCR	0.20680 (0.10805–0.44925)		1.43000 (0.10985–4830.25540)	.138
*CD24*	pCR	21.31800 (4.611100–152.20555)	.347	8.71800 (0.25955–28.33245)	.080
	No pCR	0.00880 (0.00025–158.76605)		111.73990 (4.60905–11208.7821)	.500
miR590-3p	pCR	0.15125 (0.33875–0.22475)	.085	0.95865 (0.00757–10.44982)	.465
	No pCR	0.92850 (0.17580–13.29670)		0.31490 (0.12855–8.14630)	.893
miR599	pCR	1.38130 (0.07960–169.03870)	.917	181.77370 (0.15540–855.39930)	.500
	No pCR	0.43710 (0.08275–6.25930)		1.41030 (0.266350–9.74585)	.225
miR399-3p	pCR	0.58560 (09140–4.03130)	.251	0.97130 (0.01360–67.50200)	.225
	No pCR	3.24450 (0.24985–17.86485)		0.86330 (0.07730–6.42245)	.225

pCR = pathologic complete response, *P*^1^ = *P* values indicate the difference in pretreatment level of parameters between patients with pathologic complete response and nonresponders, *P*^2^ = *P* values indicate the difference between pre- and posttreatment levels of parameters in patients with pathologic complete response and nonresponders.

**Table 7:** The pre- and posttreatment expression levels of *CD44*, *CD24*, miR590-3p, miR599, and miR399-3p by pathologic response in TN subtype of breast carcinoma.

TN	Response to therapy	Pretreatment levels median (IQR)	*P* ^1^	Posttreatment levels median (IQR)	*P* ^2^
*CD44*	pCR	1.18100 (0.00710–280.13920)	.909	0.00470 (0.00340–0.59100)	.063
	No pCR	1.48450 (0.04210–7.56850)		0.00160 (0.00010–0.04070)	.109
*CD24*	pCR	0.32130 (0.51200–129.06910)	.569	0.10310 (0.04810–0.27400)	.091
	No pCR	0.36150 (0.00100–1.60440)		0.04880 (0.00430–0.23040)	.285
miR590-3p	pCR	0.296550 (0.15840–1.18465)	.739	1.00255 (0.00157–6.00997)	.463
	No pCR	4.362050 (0.15240–6.46300)		0.28055 [0.11392–0.37922)	.180
miR599	pCR	0.18170 (0.06700–3.45810)	.569	4.0000 (0.032800–8.05560)	.499
	No pCR	5.13370 (0.45100–237.20650)		7.2100 (2.21910–29.04060)	1.000
miR399-3p	pCR	0.51190 (0.38800–1.96430)	.305	0.01790 (0.00150–2.31980)	.735
	No pCR	0.57200 (0.53000–54.34200)		0.01790 (0.00660–4.90410)	.593

pCR = pathologic complete response, *P*^1^ = *P* values indicate the difference in pretreatment level of parameters between patients with pathologic complete response and nonresponders, *P*^2^ = *P* values indicate the difference between pre- and posttreatment levels of parameters in patients with pathologic complete response and nonresponders, TN = triple negative.

We could not find any statistically significant relationship between the expression level of the *CD44* gene and OS or DFS.

### 3.2. The expression of *CD24* gene at baseline was significantly higher in patients responsive to the therapy than in nonresponders

Similar to the *CD44* gene, there was a statistically significant difference in pre- and posttreatment levels of *CD24* (*P* = .001) (Table 2). When we evaluated breast cancer subtypes separately, we observed a significant decrease in ER + and TN groups (*P* = .002 and *P* = .047, respectively) (Table 3). The expression levels of *CD24* in initial biopsies were significantly lower in nonresponsive patients than in responders (*P* = .044). Pre- and posttreatment biopsies significantly differed in patients with pCR (*P* = .001) (Table 4). Similarly, in ER + group, there was a significant decrease in *CD24* level in patients with pCR (*P* = .008) (Table 5). Patients with residual tumors had significantly decreased initial expression of *CD24* compared to the patients with no residual tumors (*P* = .044). The levels of the *CD24* gene were downregulated in patients with high-stage tumors, LN metastasis, distant metastasis, and recurrence. However, the difference was insignificant.

The levels of *CD24* were downregulated in cerbB2-positive tumors in initial biopsies. There was a significant decline in the levels of *CD24* gene in the cerbb2-negative tumors after therapy (*P* = .006) Similarly, *CD24* expression was significantly decreased in hormone receptor positive tumors after chemotherapy (*P* = .002).

*CD44* levels were higher than *CD24* levels in initial biopsies. *CD44* and *CD24* gene expression levels in pretreatment biopsies were significantly correlated. No parameter showed a statistically significant difference between the initial biopsies of low*CD44*/high*CD24* tumors and high*CD44*/low*CD24* cancers. Lastly, we could not find any significant correlation between the expression of the *CD24* gene and OS or DFS in univariate analysis.

### 3.3. The expression levels of miR590-3p in pretreatment biopsies differed significantly in patients responsive to the therapy compared to nonresponders

The level of miR590-3p was significantly increased after treatment compared to their previous counterparts (*P* = .04) (Table 2). The pretreatment levels of miR590-3p were significantly higher in nonresponsive patients than in responsive ones (*P* = .014) (Table 4). Similarly, posttreatment miR590-3p levels also statistically differed in patients with pCR compared to pretreatment levels (*P* = .011) (Table 4).

Considering clinical subtypes, we observed a significant increase in miR590-3p levels only in the ER + group (*P* = .017) (Table 3). In ER + subgroup, the level of miR590-3p in patients with pCR significantly increased after NAC (*P* = .017) (Table 5).

**Figure 1.**
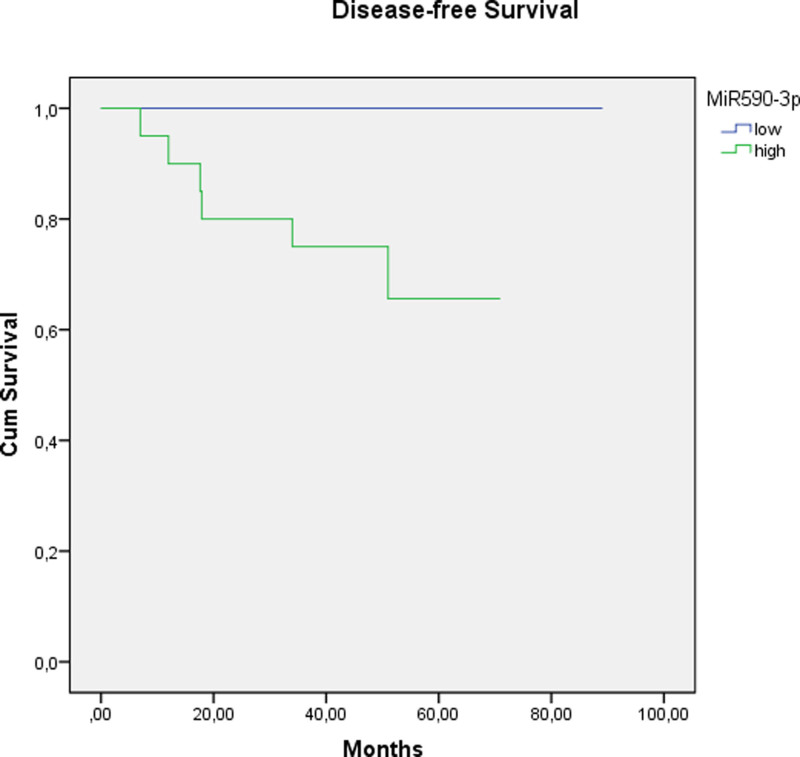
The expression level of miR590-3p at initial biopsies was significantly correlated with disease-free survival in all terms (*P* values for short, medium, and long terms, respectively; *P* values: .009, .008, and .007).

The pretreatment levels of miR590-3p in recurrent cases were significantly higher than in nonrecurrent cases (*P* = .009) in breast cancer patients. Although its limited number, in the TN group, miR590-3p was significantly higher in patients with LN metastasis and distant metastasis (*P* = .019 and .019, respectively). In our study, there was a statistically significant correlation between the levels of miR590-3p and DFS, but not with OS (Table 8). Univariate analysis revealed that high levels of miR590-3p were associated with worse DFS (Fig. 1).

**Table 8:** Univariate analysis of miR590-3p, miR599, and miR399-3p for disease-free survival.

Pretreatment levels	*P* value	Hazard ratio	CI 95%
miR590-3p	.04	1.001	[1.000–1.001]
miR599	.001	1.010	[1.004–1.016]
miR399-3p	.016	1.001	[1.000–1.001]

### 3.4. The expression levels of miR599 were higher in recurrent cases than in nonrecurrent cases in pretreatment biopsies

The miR599 levels did not significantly differ pre- and posttreatment samples. However, we observed that the pretreatment level of miR599 was lower in patients with pCR in ER + group (*P* = .017) (Table 5).

**Figure 2.**
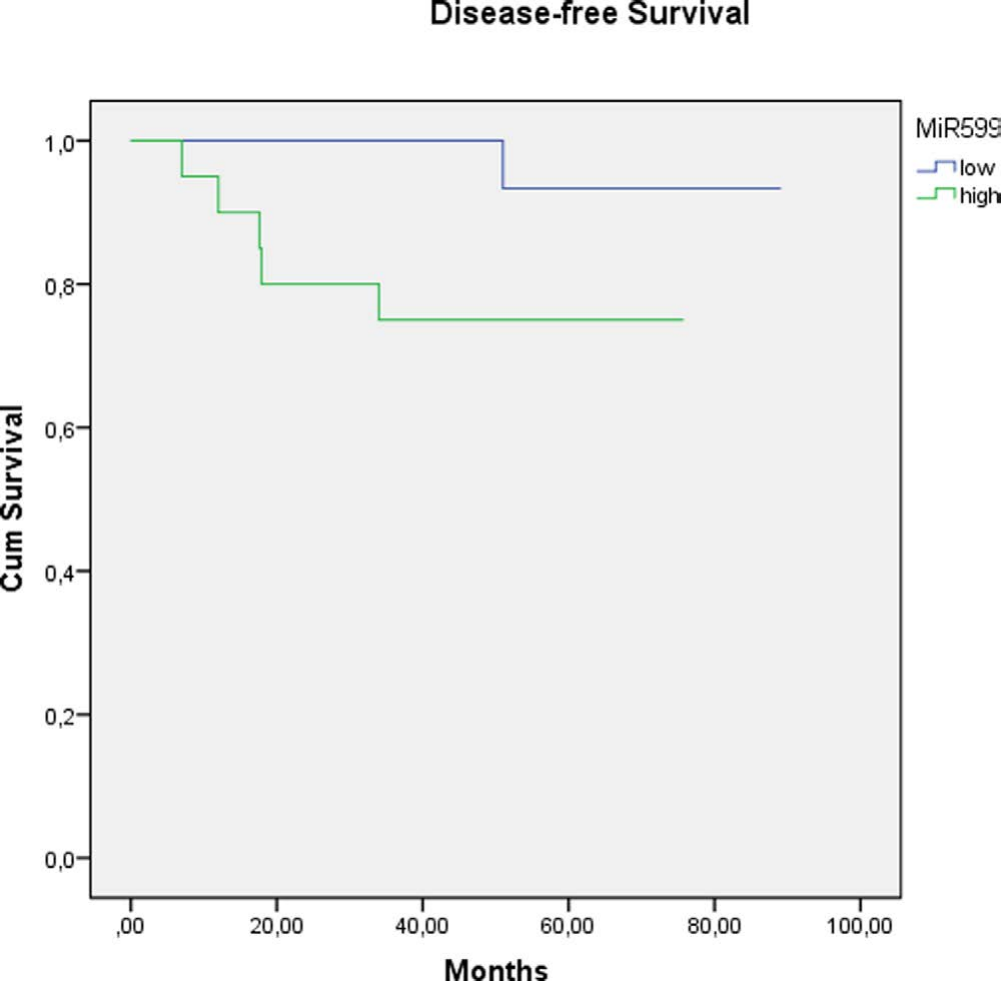
The expression level of miR599 at initial biopsies was significantly correlated with disease-free survival in the short and medium terms but not for the long term (*P* values: .03, .04, and .05, respectively).

Similar to miR590-3p, there was a significant difference between the levels of miR599 in patients with recurrence compared to those with no recurrence at baseline (*P* = .004). Moreover, miR599 levels in pretreatment biopsies were positively correlated with the stage of the disease (*P* = .034). In addition, it was found that the high levels of miR599 were significantly associated with DFS in the short and medium terms (Fig. 2). There was no significant relationship between OS and miR599 levels. Univariate analysis revealed that high levels of miR599 were associated with worse DFS rates (Table 8).

### 3.5. The expression levels of miR399-3p were significantly higher in patients with no complete pathologic response than responders in pretreatment biopsies

**Figure 3.**
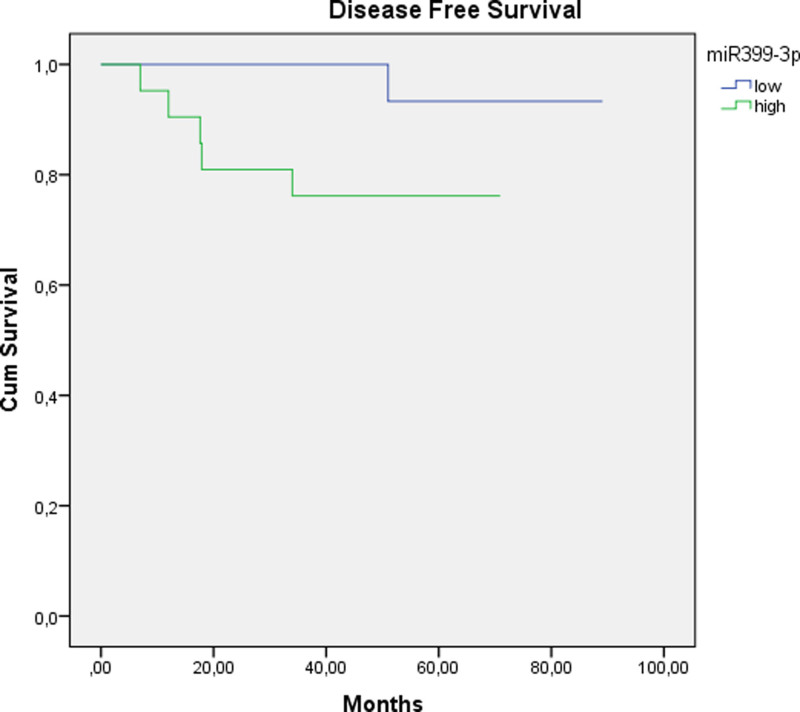
The expression level of miR399-3p at initial biopsies was significantly correlated with disease-free survival in the short and medium terms but not for the long term (*P* values: .04, .05, and .07, respectively).

The levels of miR399-3p showed no significant difference between pre- and postreatment biopsies. There was a significant difference between pretreatment miR399-3p levels of responsive patients and nonresponders (*P* = .027) (Table 4). There was no significant correlation between miR399-3p levels and OS. There was no significant difference between the pretreatment levels of miR399-3p in patients with recurrences and nonrecurrents (*P* = .09). However, univariate analysis revealed that high levels of miR399-3p were associated with worse DFS rates (Table 8 and Fig. 3). Multivariate analysis revealed no significant association.

## 4. Discussion

This study demonstrated that pretreatment levels of the *CD24*, miR590-3p, and miR399-3p differed significantly between patients with pCR to therapy and nonresponders in breast carcinoma. This study showed that the increased levels of miR590-3p, miR599, and miR399 in initial biopsies are associated with poor DFS.

*CD44* is an integral membrane protein used as a CSC marker in many cancers, including breast cancers. There are controversial findings about the relationship between *CD44* levels and pathological parameters. Some studies reported no association between *CD44* levels and LN status, recurrence, distant metastasis, or pathological response to treatment.^[11–13]^ Some studies found that high expression of *CD44* was associated with aggressive tumor-related features and decreased survival rates.^[14]^ Conversely, *CD44* expression was reported as decreased in patients with LN metastasis.^[15]^ Similar to the former studies, the expression level was significantly higher in patients with high-stage tumors, LN metastasis, and distant metastasis in our study. However, on the contrary, *CD44* was downregulated in initial biopsies of patients with recurrences and without pCR. *CD44* is reported to have a dual role in cancer progression. In vitro studies have shown that *CD44* overexpression is associated with invasiveness; nevertheless, *CD44* null mutation has been linked to a protective effect against breast carcinoma.^[16]^ Therefore, the role of *CD44* is unclear, yielding contradictory results. Following the literature, we found controversial results in this study. This dual nature of *CD44* can be explained by *CD44* having different isoforms, and the expression of these variably spliced isoforms results in changes in *CD44*-mediated biology; alternative expression has been linked to increased metastatic activity. Additionally, the tumorigenicity of breast and other cancer types can be influenced by changes in the expression of miRNAs that regulate *CD44* expression.^[17]^

The current study demonstrated that the mRNA expression level of *CD44* was decreased after NAC. Levels of *CD44* in the ER + and TN groups were also significantly declined. The literature reports that overexpression of *CD44* is related to the TN subtype of breast cancer and the correlation between expressions of cerbB2 and *CD44.*^[18]^ Similarly, we found a positive correlation between cerbB2 and *CD44* levels. Given the correlation between cerbB2 and *CD44*, this difference might be a result of cerbB2 negativity in these groups. *CD44* level was upregulated in the patients with pCR in initial biopsies, and it was significantly reduced after NAC. Although there was no correlation between ER positivity and *CD44* levels in our study, the patients with pCR in ER + group showed a significant decrease after therapy. The possible crosstalk between ER positivity and *CD44* gene expression remains unknown and needs to be further evaluated. In a recent study, immunohistochemical expression of *CD44* was found to be related with aggressive behavior and poor therapeutic response in breast cancer.^[19]^ Similar to that, our findings suggest that upregulation of *CD44* was associated with worse prognostic factors such as LN metastasis, distant metastasis, and high-stage. In contrast, patients with pCR also had upregulation in their initial biopsies in our study. Cancer treatments target the growing population of tumors and mostly underestimate the importance of tumor niche. Tumors can recur and metastasize despite pCR.^[20,21]^ The tumor microenvironment includes several factors facilitating tumor development.^[22]^ Mesenchymal stem cells in the tumor microenvironment are reported to enhance breast CSC proliferation and promote metastasis.^[23]^ Given the regulating properties of *CD44* and *CD24*, they might enhance malignant transformation in microenvironment.^[24]^ Parallel to that, expressions of *CD44* are reported as similar in both cancerous cells and adjacent noncancerous cells.^[25]^ Because of the importance of the tumor microenvironment, the tumor bed in pCR patients can also reflect many changes. Moreover, the decline was observed in both responders and nonresponders in our study. This may be explained by the dual character of *CD44* too. It is also postulated that it can have different behavior in different tumors or different stages of tumors via using various pathways.^[16]^ Besides *CD44*’s dual role in tumor biology, our small sample size could contribute to controversial findings in current study.

*CD24 + *cells have a high metastatic capacity and higher tumorigenic potential in vitro studies.^[26,27]^ The overexpression of *CD24* is related to low OS.^[28]^ However, Ricardo et al found no relationship between *CD24* expression and pathological parameters.^[15]^ In vitro studies showed that *CD44+*/*CD24 + *tumor cells and *CD44+*/*CD24‐* tumor cells differed in their stemness properties. The *CD24 + *cells have a higher renewal capacity, but *CD24‐* cells have a more enhanced clonogenic capacity.^[29]^ This might cause conflicting results in the literature.

In a recent study, increased immunohistochemical expression of *CD24* was found in patients with poor response.^[30]^ In contrast to that, our study found a significant downregulation in pretreatment *CD24* expressions in patients with no pCR, indicating poor prognosis. The responders had upregulated *CD24* levels.

An in vitro study has been shown that *CD24* increased after docetaxel treatment but decreased or unchanged after doxorubicin treatment.^[31]^ Despite the lack of knowledge about therapy regimens, we observed a decline in posttreatment levels of *CD24* in all patients regardless of pCR. The decrease was also significant in the ER + and TN groups. The decreased levels of *CD24* after therapy might be associated with the contribution of *CD24* to therapy resistance. In our study, half of the recurrent cases had pCR. According to recent literature, anticancer therapy stresses tumor cells, but CSCs can avoid this pressure because they are intrinsically resistant to chemotherapeutics. In addition, differentiated cancer cells may acquire stem-like characteristics due to this selective pressure.^[32]^ Consistently, the loss of *CD24* in breast cancer cells is reported to be related to therapy resistance by inducing stemness properties.^[33]^ These findings suggest *CD24* might be an earlier parameter for predicting therapy resistance. Supporting that, *CD24* is reported as a better target candidate to overcome therapy resistance.^[27]^

Accordingly, although the difference was not significant, we observed that decreased *CD24* expression levels were related to poor prognostic parameters, including stage, LN positivity, distant metastasis, and recurrence. The loss of cerbB2 expression is reported to be related to poor prognosis in breast cancer patients.^[34]^ Consistently, we found a lower expression of *CD24* in cerbB2-positive tumors.

Kim et al reported that overexpression of *CD24* is related to decreased OS and DFS and is a poor prognostic factor in hormone-positive tumors.^[35]^ However, we could not find any relationship between *CD24* expression and OS or DFS in these patients.

In our study, we found *CD44* levels were higher than the levels of *CD24* in initial biopsies. Similarly, Ricardo et al reported that most tumors have higher levels of *CD44* expression and lower levels of *CD24* expression.^[15]^ A high *CD44*/*CD24* ratio was reported to be related to tumor stemness, cell proliferation, and tumorigenesis.^[24]^ A high ratio of *CD44*/*CD24* was also found to be associated with shorter OS in breast carcinoma.^[11]^ However, we could not find any significant relationship between *CD44*/*CD24* ratio and any other parameter.

Chemotherapeutics induce phenotypic switching in CSCs. Microenvironmental changes, some signaling pathways, epigenetic events, and the modulation by microRNAs can contribute to this plasticity.^[32]^ MicroRNA controls gene expressions by inducing or inhibiting them. Several microRNAs have been identified in cancer progression by regulating stem cell markers and metabolism.^[16]^ Limited studies showed that miR590-3p regulates proliferation in breast cancer and can participate in tumorigenesis via signaling pathways.^[36,37]^ Although miR590-3p is reported to promote proliferation and metastasis in colorectal carcinomas^[38]^; and upregulation of miR590-3p is associated with anticarcinogenic properties and inhibits metastasis and proliferation in breast carcinomas.^[39]^ Limited studies reveal contradictory results. In our study, patients with elevated levels of miR590-3p did not show pCR after NAC. miR590-3p was significantly higher in patients with recurrences and LN and distant metastasis, especially in the TN group. Despite of the limited number of patients with pCR in this group, these results represent very crucial preliminary clues for future studies. The patients with upregulated miR590-3p levels in initial biopsies have shorter DFS. All these findings suggest high levels of miR590-3p indicate poor prognosis. Moreover, we found that miR590-3p levels are significantly increased with therapy. The significant increase in ER + group suggests a potential relationship between miR590-3p and hormone receptors which remains unknown and needs further research. The increase might be due to the contribution of miR590-3p to therapy resistance like *CD24*, as miR590-3p promotes radiotherapy resistance in colorectal cancers.^[40]^ We think that miR590-3p levels might indicate poor pathologic response in breast cancer patients and its further elevation might be a predictive sign of therapy resistance.

The downregulation of miR599 is reported as a marker of poor prognosis in cervical cancer and malignant melanomas.^[41,42]^ Overexpression of miR599 is associated with metastasis in colorectal cancers.^[43]^ However, it is suppresses invasion and metastasis in breast cancer cell lines.^[44]^ On the contrary to the latter, we found that upregulation of miR599 is related to advanced stage and recurrence. Similarly, we found increased pretreatment levels of miR599 in patients with no pCR and the increase was significant in the ER + group. This novel finding might uncover a new research area in breast cancer. We suggest that the higher levels of miR599 in initial biopsies might predict nonresponsiveness to the therapy in ER + breast carcinoma. The patients with initially higher levels of miR599 have shorter DFS in the short and medium terms. miR599 might predict recurrences and poor response to therapy.

Data is limited about miR399-3p and its role in cancer. It is known that metabolic pathways have been changed in cancer cells. Limited studies show that miR399-3p has a role in the sulfur mechanism and is upregulated under sulfur deprivation.^[45]^ A recent study reported that sulfur-poor diet can be protective against breast carcinoma.^[46]^ Therefore, we can assume that miR399-3p can have a potential role in breast cancer. We found downregulation of miR399-3p in initial biopsies in patients responsive to the therapy. Our results showed that high levels of miR399-3p might be a predictive marker for poor therapy response and worse disease-free survival rates.

Although this study contributes novel and important data to the literature, it has some limitations. First, we conducted this study with only a small group of breast cancer patients with different molecular subtypes. Although we achieved significant results for ER + patients, other subgroups included a small number of patients with pCR. Future studies with larger cohorts, especially for HER2 + and TN breast cancers, are needed to validate these preliminary observations. Secondly, we performed qPCR analysis and couldn’t correlate immunohistochemistry. The *CD44* and *CD24* genes might have different mRNA and protein levels. Future studies concerning both methods might clarify these discrepancies.

## 5. Conclusion

Genes and miRNAs regulating CSC properties and related pathways are promising because they can be novel therapeutic targets and predictive biomarkers for prognosis and therapy response. Our study suggests that the downregulation of the *CD24* and upregulation of miR590-3p might indicate poor therapy response, and their further decrease and increase after therapy might be early and poor prognostic factors for therapy resistance in breast carcinoma. Moreover, upregulation of miR590-3p, miR599, and miR399-3p might be a marker of poor prognosis with shorter DFS.

## Author contributions

**Conceptualization:** Tuba Dilay Kökenek Ünal, Müzeyyen Burcu Kaplan Yilmaz, Olçay Kandemir.

**Data curation:** Samet Yora, Serpil Taheri.

**Formal analysis:** Tuba Dilay Kökenek Ünal, Müzeyyen Burcu Kaplan Yilmaz, Olçay Kandemir, Samet Yora, Serpil Taheri.

**Funding acquisition:** Tuba Dilay Kökenek Ünal, Müzeyyen Burcu Kaplan Yilmaz, Olçay Kandemir.

**Investigation:** Samet Yora, Serpil Taheri.

**Methodology:** Müzeyyen Burcu Kaplan Yilmaz, Olçay Kandemir.

**Project administration:** Tuba Dilay Kökenek Ünal, Müzeyyen Burcu Kaplan Yilmaz, Olçay Kandemir.

**Resources:** Tuba Dilay Kökenek Ünal, Müzeyyen Burcu Kaplan Yilmaz, Samet Yora, Serpil Taheri.

**Supervision:** Tuba Dilay Kökenek Ünal, Müzeyyen Burcu Kaplan Yilmaz, Olçay Kandemir.

**Validation:** Tuba Dilay Kökenek Ünal, Müzeyyen Burcu Kaplan Yilmaz.

**Writing—original draft:** Tuba Dilay Kökenek Ünal, Serpil Taheri.

**Writing—review & editing:** Tuba Dilay Kökenek Ünal, Serpil Taheri.
